# Extreme Events, Entrepreneurial Start-Ups, and Innovation: Theoretical Conjectures

**DOI:** 10.1007/s41885-021-00089-0

**Published:** 2021-07-10

**Authors:** Thomas Gries, Wim Naudé

**Affiliations:** 1 Department of Economics, University of Paderborn, Paderborn, Germany; 2 Cork University Business School, University College Cork, Cork, Ireland; 3 RWTH Aachen University, Aachen, Germany

**Keywords:** Entrepreneurship, Innovation, COVID-19, Extreme events, Development

## Abstract

In light of the COVID-19 pandemic, we scrutinize what has been established in the literature on whether entrepreneurship can cause and resolve extreme events, the immediate and long-run impacts of extreme events on entrepreneurship, and whether extreme events can positively impact (some) entrepreneurship and innovation. Based on this, we utilize a partial equilibrium model to provide several conjectures on the impact of COVID-19 on entrepreneurship, and to derive policy recommendations for recovery. We illustrate that while entrepreneurship recovery will benefit from measures such as direct subsidies for start-ups, firms’ revenue losses, and loan liabilities, it will also benefit from aggregate demand-side support and income redistribution measures, as well as from measures that facilitate the innovation-response to the Keynesian supply-shock caused by the pandemic, such as access to online retail and well-functioning global transportation and logistics.

## Introduction

The economic consequences of the COVID-19 pandemic that broke out at the end of 2019 are the most destructive since the Second World War (World Bank 2020). This is as a result of the extensive non-pharmaceutical interventions (NPIs) - or “lockdowns” - that most countries implemented in order to curtail the spread of the SARS-CoV-2 virus and shield health systems from being overwhelmed (Ferguson et al. 2020; Hale et al. 2020). Throughout the world, this has severely curtailed entrepreneurship on the whole, notwithstanding the fact that the crisis created opportunities for some entrepreneurs in certain industries (Zahra 2020) and that start-ups rates have increased in certain countries (Romei 2020). As discussed by Naudé (2020a) the impacts will be of both a shorter and longer-term nature. Over the shorter term start-up rates for businesses based on opportunity-motivations may decline^1^ and business failure rates rise, ceteris paribus, due to the the business cycle impact of a world economy that has gone into a sudden and deep recession.^2^ Over the longer-term COVID-19 could have permanent impacts on entrepreneurship and contribute to a “prolonged recession” (Fairlie 2020, p.12). The danger therefore is that the COVID-19 pandemic will intensify the secular decline in entrepreneurship that is already noticeable in most advanced economies (Decker et al. 2016; Hopenhayn et al. 2018; Naudé 2020b).

The search for policy responses to mitigate the adverse consequences of lockdowns, and to reduce the possible long-term fallout on entrepreneurship and the innovativeness of economies, will benefit from the availability of theoretically grounded and consistent models that can aid understanding of how extreme events^3^, such as COVID-19 and the 2009-2010 global financial crisis for example, are structurally related to entrepreneurship. In 2011, in the immediate aftermath of the 2009-2010 global financial crisis, Gries and Naudé (2011, p.2) made the point that the relationship between entrepreneurship and extreme events were still poorly understood. In the meantime, there has been a growing literature on entrepreneurship in “difficult” contexts or extreme events, such as in conflict (e.g. Brück et al. 2013, Desai et al. 2013), genocide (e.g. Stel and Naudé 2016), social crises (e.g. Munoz et al. 2020), refugee-ship (e.g. Desai et al. (2020) and natural disasters (e.g. Crespo Cuaresma et al. 2008, Boudreaux et al. 2019). The relationship between entrepreneurship and “crises” more generally, including the concept of “crisis” are examined in Doern et al. (2019). Much of this useful literature tend to be empirical and descriptive, so that the need for theorizing remain. Good theorizing precedes empirical description and provide explanations, as distinct from descriptions. It is the essence of scientific thinking. As Deutsch (2011, p.4) put it “scientific theories are not ‘derived’ from anything [...] They are guesses - bold conjectures”.

In this paper we provide some bold conjectures, using a novel theoretical model, to explain the impacts of extreme events, and specifically the impact of a pandemic such as COVID-19, on entrepreneurship. We do so by selectively reviewing the current state of the art, and departing from our own earlier work on the impact of the global financial crisis on entrepreneurship - see Gries and Naudé (2011). Moreover, we argue that the particularities of the COVID-19 crisis need to be taken explicitly into account, and that these are different from other extreme events. In particular, we consider the fact that, as discussed by Brinca et al. (2020) and Guerrieri et al. (2020), that COVID-19 is a “Keynesian” supply-shock, that is a supply-shock which causes a reduction in aggregate demand whilst keeping a part of the economy locked down and unable to respond in terms of supply to expansionary fiscal policy measures. Our model’s comparative statics shows that entrepreneurship recovery after COVID-19 will in general benefit from aggregate demand-side support measures, but even more pertinently if combined with additional measures such as a direct subsidies for start-ups, firm revenue losses and loan liabilities. Entrepreneurship will also benefit from a redistribution of income to the extent that it shifts more income to consumers who have a higher propensity to consume. Given that expenditure shifts will increase opportunities for some entrepreneurs in certain industries (see Zahra 2020), and given that there may be an innovation-response to this as well as to the Keynesian supply-shock caused by the pandemic, the total effect of all of this may even be to result in an increase in start-up rates over the short-term.

The rest of the paper is structured as follows. In section we summarize the relevant literature on the impacts of extreme events on entrepreneurship, and in particular start-ups and innovation. Then, in “Theoretical Conjectures” we provide a simple but novel model to trace these impacts analytically and take into account the demand-side nature of the shock from COVID-19. Section “Impact of an Extreme Event: Comparative Statics” describes the comparative statics of the model from which policy implications can be drawn. Section “Concluding Remarks” concludes.

## Relevant Literature

In this section we review the literature that has dealt with the impact of extreme events on entrepreneurship, as well as the literature that has tried so far to measure the impact of COVID-19 on start-ups and innovation. We believe that there is much to be learned from previous extreme events, not least the fact that the COVID-19 crisis is, from both its health and economic impacts, whilst hugely significant, not the worst crisis the world has overcome in the past century. Based on, and adding to these strands of literature, we then propose a simple (partial equilibrium) model in “Theoretical Conjectures” that will be able to explain the salient features of crises and entrepreneurship, and offer analytical tractability to explore policy responses, which we will illustrate in “?? ??”.

We summarize the literature from the points of view of whether and how entrepreneurship can cause or resolve major crises (“?? ??”), how entrepreneurs are affected and cope in the aftermath of a crises (“?? ??”), and whether and how entrepreneurship and innovation are affected over the long-run (“?? ??”).

### Can entrepreneurship cause and resolve extreme events?

Most extreme events, especially natural disasters, tend to be exogenous shocks as far as entrepreneurship is concerned (Brück et al. 2011). However, following the distinction between productive, unproductive and destructive entrepreneurship (Baumol 1990), there have been a number of papers that have explored how the incentive structure of society can, via the choices of entrepreneurs, lead it into macro-economic or political crisis, and/or into violent conflict and high rates of crime, see e.g. Antony et al. (2017) or Desai et al. (2013). Stel and Naudé (2016) give an account of the complicity of entrepreneurs in genocides and mass atrocities, while Myburgh (2017) documents how “government capture” by entrepreneurs caused a political and economic crisis of substantial magnitude in South Africa.

As far as the 2009-2010 global financial crisis is concerned, entrepreneurial culpability has been raised by Naudé (2010) who ascribed the crisis to inappropriate incentives towards excessive risk-taking and moral hazards due to banks having become “too-big-to-fail” - in essence arguing that entrepreneurial ability was disproportionately attracted by these incentives and moral hazards into the financial sector (see also Johnson and Kwak (2011)). Here, many with high entrepreneurial ability engaged not in productive entrepreneurship first and foremost, but rather in “innovation activity aimed purely at regulatory arbitrage, excessive leverage, and the so-called ‘search for yield’ which is just a polite way of describing the practice of shifting assets to riskier and illiquid ones” (Acharya et al. 2009, p.111).

Thus, entrepreneurs can certainly cause and contribute to extreme events. But what about natural disasters specifically? While these are mostly exogenous (one may however argue that extreme weather events may be partly due to anthropomorphic climate change) and not caused by entrepreneurship, entrepreneurial responses may increase their likelihood. For example in the case of the COVID-19 pandemic, human land use patterns, including the operation of so-called “wet markets” have been implicated (Aguirre et al. 2020), and are considered to make the future outbreak of pandemics likely^4^ (Gibb et al. 2020).

While entrepreneurs can cause, contribute and even raise the likelihood of an extreme event, they can also be instrumental in coping with, resolving or leading recovery from such an event. Doern et al. (2019, p.405) discuss the role of entrepreneurs as agents embedded in their local community, where they provide these communities “with critical resources in the aftermath of a crisis, in the form of products and services, or donating materials, money and time to victims.” Entrepreneurs can promote peace and reconstruction following violent conflicts by creating jobs, vehicles for social mobility and deployment of former combatants, and even by providing goods of a public or quasi-public nature, such as banking services and infrastructure (Naudé 2009; Munoz et al. 2020). Another role that entrepreneurs can play in recovering from an extreme event, is in driving economic growth and being the causal factor behind an upswing in the business cycle (Koellinger and Thurik 2012). Often this is facilitated by a declining interest rate (as after the 2009-2010 global financial crisis) due to a rise in household precautionary savings and flights to “safety” (Caballero and Farhi 2018). This enables investment in new capital and technology, often to replace that made obsolete or destroyed by the crisis, which is, as we show in the next subsection and in our theoretical model, why extreme events are not infrequently associated with consequent growth and innovation episodes^5^.

### How are entrepreneurs affected by extreme events?

The COVID-19 pandemic, being an extreme event on a global scale, suggests that such events can severely curtail entrepreneurship on the whole (Bartik et al. 2020; Fairlie 2020), notwithstanding the fact that it may create opportunities for some entrepreneurs in certain industries (Zahra 2020) and stimulate creative destruction (Ionescu-Somers 2020). Before discussing some of the positive and long-run impacts, we will briefly summarize what is known about the immediate impacts of an extreme event like COVID-19 on entrepreneurship and innovation. In the discussion and theoretical modelling that follow, we focus on the crisis-impacts on new venture start-ups and innovation, and their nature and determinants. We do not focus on the impact on entrepreneurs’ subjective and mental well-being and their coping mechanisms. These are important, but fall outside the scope of our paper : the interested reader is referred to Doern et al. (2019), Giones et al. (2020) and Ahlheim et al. (2020) for a discussion of these.

#### Immediate negative impacts

Extreme events often manifest as a supply-shock to the economy: for instance a flood will destroy infrastructure and access to labor, which would result in a sudden drop in output supplied. Similarly a terrorist attack could destroy infrastructure and disrupt supply chains, reducing output supplied. Most often these impacts are of a short-lived nature (Boudreaux et al. 2019), and can be (partly) mitigated by insurance (Borensztein et al. 2017). COVID-19 also delivered a supply-shock. It lead to factory and shop closures, disruptions in supply chains and logistics (Singh et al. 2021), restrictions in air travel (Nakamura and Managi 2020) and a sharp drop in hours worked and output supplied (Rio-Chanona et al. 2020).

But COVID-19 is more than a supply-shock: due to the fact that its main economic impact came through NPIs - lockdown measures^6^ - and due to the large degree of uncertainty as to the duration of such measures, which have been unique in living memory, it also resulted in a demand-side shock, as for instance Brinca et al. (2020) and Rio-Chanona et al. (2020) discuss. The demand-shock has to large extent been catalysed by the reduction in new ventures, by the increase in firm exits, and in the increase in unemployment. It has been termed a Keynesian supply-side shock, and its “basic intuition is simple: when workers lose their income, due to the shock, they reduce their spending, causing a contraction in demand” (Guerrieri et al. 2020, p.2). The contraction in demand could even outweigh the supply contraction, for instance estimates for the USA suggest that the lockdown policies will result in a decline in average consumption demand of 22 percent in the first year (Eichenbaum et al. 2020). Andersen et al. (2020) estimates a similar sized declined - around 27 percent - in Denmark. And according to Mandel and Veetil (2020) the aggregate effect of lockdowns globally could amount to 33 percent of global GDP. It should be added here that the reduction in consumption following the outbreak of COVID-19 is not solely due to the formal (legal) lockdown restrictions: due to uncertainty (Andersen et al. 2020), fear of contagion (Aum et al. 2020), as well as in order to avoid social stigma (Kurita and Managi 2020; Katafuchi et al. 2020), people will reduce their mobility and consumption out of their own volition.

As could be expected, the Keynesian supply-shock had an immediate negative impact on entrepreneurship through an increase in actual and expected firm failures across countries (Saez and Zucman 2020; Bosio et al. 2020). It also affected potential new businesses through reducing the start-up rate of new firms, firms that are an important source of innovation and jobs (Sedlacek and Sterk 2020). Early evidence from the USA by Fairlie (2020) found that the number of active business owners in the country declined by 22 percent between February and April 2020. Sedlacek and Sterk (2020) found that in New York State, which was heavily impacted by COVID-19, high-propensity business applications (a measure of start-ups) declined by 50 percent in March-April 2020 compared to the same period in 2019. Preliminary evidence from many other countries suggested evidence of high rates of initial firm failures and of especially small businesses facing a precarious situation, with only enough cash reserves to tie them over for a few months (Bartik et al. 2020; Bosio et al. 2020).

**Fig. 1:**
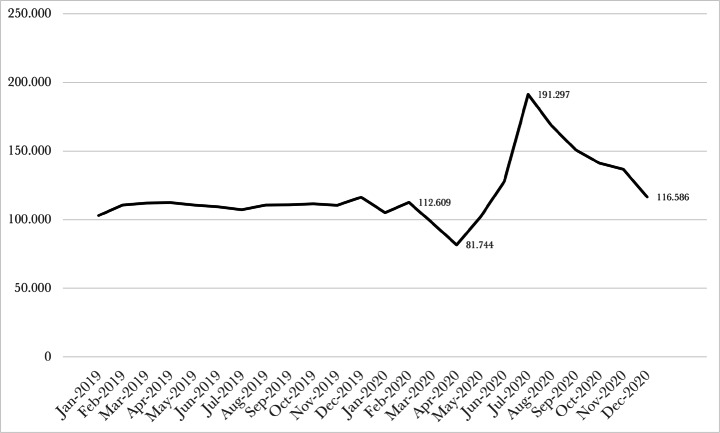
High-Propensity Business Applications (Units) in the USA, Jan 2019 - Dec 2020, Seasonally Adjusted *Data source*: USA Census Bureau, at https://www.census.gov

As we show below in Fig. 1, using data from the USA Census Bureau, (high-propensity) start-up rates in the USA indeed fell significantly in the first months of 2020, but after June 2020 started to rise sharply. These rises were also seen in other countries, such as France and Germany (Romei 2020). This means that clearly, there can also be positive impacts in terms of entrepreneurship from an extreme event. In the next sub-section we explore why this may be the case.

#### Potential positive impacts

As far the impacts of extreme events on entrepreneurship are concerned, an interesting result from the literature is that the magnitude and sign of the impact depends on the nature and magnitude of the crisis itself. For instance, as was mentioned above, extreme events can even have positive impacts on entrepreneurial dynamics as a whole. Brück et al. (2011, p.S81), using cross-section data covering 43 countries over the period 2002 to 2005 found that extreme events such natural disasters and terrorism can have a positive impact, in particular that “extreme events may encourage new businesses. The disruption of customary habits and the weakening of traditional institutions create opportunities and may change the balance of power in favor of smaller, more flexible, organizations”.

Crises will also affect some entrepreneurs more favorably than others. In the current COVID-19 pandemic for instance, Zahra, (2020, p.3) has pointed out that “many industries have experienced growth since Covid. Examples include enterprise technology services, home entertainment, AI, robotics, telemedicine, hospitals, medical equipment suppliers, e-commerce retailers, e-learning providers, courier pick-up and delivery services, cybersecurity, and sanitary product manufacturing, among many others.” This is consistent with a broader literature on the impacts of natural disasters on economic growth and TFP growth (a proxy for innovation) which have established based on cross-country empirical evidence, that many countries experience higher GDP and TFP growth after a natural disaster. A possible reason could be, as Crespo Cuaresma et al. (2008, p.214) suggest, that “disasters provide opportunities to update the capital stock and adopt new technologies, thus acting as some type of Schumpeterian creative destruction.”

We need to temper this positive view in the case of COVID-19, notwithstanding the quote from Zahra (2020) because the heterogeneity in impact is not in any way due to the heterogeneous productivity of firms, but because of the nature of lockdown measures, which essentially closed services industries, such as in hospitality, travel, events and many personal services, where physical distancing is difficult to maintain, as opposed to other industries, such as in manufacturing or transport or agriculture, where production could essentially continue with much less disruption (Bhorat et al. 2020). Guerrieri et al. (2020) illustrate that in such a case, the supply-side shock from the affected industries will spill-over to the demand for the goods from the unaffected industries (i.e. a Keynesian supply-shock), and that raising aggregate demand will impact with a smaller Keynesian multiplier than when the supply response of certain industries are in effect precluded. Hence, the Keynesian supply-shock is a relative novel feature of the COVID-19 pandemic which makes the distributional consequences and demand recovery of the economy more complex, and thus likely to be more long-lasting (see also sections 2.2.3 and 2.3 below).

However, the Keynesian supply-shock feature of the COVID-19 crisis may result in an innovation-response from entrepreneurs and hence a rise in innovation-driven start-ups. This is because the industries that are affected by the lockdown- such as in events, retail, education and others - may stimulate the creation of new ventures to support them through digital/ online business models to overcome the lockdown constraints (Romei 2020). This will however be dependent on governments providing fiscal support for households and existing firms, and measures to reduce the health impact and hence the broader uncertainty surrounding the pandemic. Furthermore, it will be dependent on global transport and logistic systems remaining open and functioning, and access to adequate ICT. Indeed, both government fiscal (rescue) packages and support, as well as the fast recovery in global value chains^7^ (see e.g. Naudé and Cameron (2021) have been noticeable during 2020. It is therefore quite likely that at least over the short-term, the last months of 2020 could have seen start-up rates increase, especially in countries where the required conditions were in place.

#### Distributional consequences

These heterogeneous impacts of crises means that they will have particular distributional consequences - creating winners and losers (Gharehgozli et al. 2020). Global pandemics in particular seem to raise inequality and market concentration. Furceri et al. (2020) shows that the Gini-coefficient, a measure of income inequality, has tended to worsen on average by 1,5 percent after a pandemics in the past. Palomino et al. (2020) estimates that as a result of the COVID-19 pandemic, that income inequality in European countries will increase between 2 and 21 percent.

Another indicator of inequality, as well as of the degree of contestability of markets, is the extent of market concentration. There is widespread agreement that one impact of the COVID-19 pandemic (as of the 2009-2010 global financial crisis) will be to raise market concentration. One indication is the growing market capitalization of large global platform firms. This effect will not be unusual for a pandemic. In the 14th century for instance, the Black Death boosted the market dominance and wealth of a few well-positioned incumbents. As Russell and Parker (2020) describe, after the Black Death market concentration increased and the influence of big business on government grew, an outcome also likely in the present crisis, since “while small companies rely upon government support to prevent them collapsing, many others, mainly the much larger ones involved in home delivery, are profiting handsomely from the new trading conditions.”

While global inequality actually declined^8^ after the global financial crisis of 2009-2010 (Milanovic 2020), this may not be the case after the COVID-19 pandemic, not only due to advanced economies being better resilient in having recourse to ICT infrastructure for online commerce (Naudé 2020a), but also better recourse to financial resource to reduce unemployment and business failures, and to “update” their capital stock and invest in new start-ups (Crespo Cuaresma et al. 2008; Brück et al. 2011). Another way in which the COVID-19 pandemic will likely raise inequality between countries, is, as Gries and Naudé (2011) noted in the case of the global financial crisis, is that GDP growth and entrepreneurs in developing countries are more dependent on international trade than in advanced economies where internal markets tend to be larger, so that the sharp reduction in travel, mobility and the delays throughout global supply chains, may have a dis-proportionally negative impact on (international) entrepreneurship in developing countries. As Gries and Naudé (2011) noted, “a major reason for entrepreneurs to innovate is to be able to compete internationally [...] a global shock removes this incentive.”

Whereas moderate inequality could be an incentive for risk-taking and entrepreneurship, high levels of inequality is on balance, bad for entrepreneurship and innovation. High inequality lead to changes and declines in consumption demand which will affect the size of the market and entrepreneurial opportunities; higher inequality is also associated with greater social instability and uncertainty, which in turn depresses entrepreneurial investment (Zweimueller 2000; Doucouliagos 2017). With start-up funding falling, and most SMEs only able to cover a few months costs in the face of a COVID-19 lockdown (Bartik et al. 2020), it will be the case, as in the 2009-2010 global financial crisis, that the more wealthy entrepreneurs will be more able to start a firm during the crisis (see also Naudé and MacGee 2009). This itself can worsen wealth inequalities which in turn can further reduce start-ups and moreover reduce social mobility and the ability of entrepreneurship to offer a pathway out of poverty (Marinoni and Voorheis 2019). As Mesnard and Ravallion (2005, p.3) point out “greater wealth inequality implies that fewer potential entrepreneurs are able to finance start-up capital”. Thus, what may be expected during the COVID-19 crisis is that credit constraints become more binding relative to new venture creation (start-ups) so that the start-up rate drops, leading further to declines in the average size of firms, and further disparities between large and small firms, and greater concentration.

Our conclusion from this literature is that on balance, extreme events affect entrepreneurship negatively, even though some entrepreneurs benefit, and crises may be followed by growth and innovation episodes. There may even be over the short-term an increase in innovation-driven start-up rates as entrepreneurs respond creatively to overcome the constraints imposed by the extreme event. The long-run implications of the these consequences will be discussed in more detail in the next subsection.

### What are the long-run implications for entrepreneurship and innovation?

The concern is that over the longer-run there could be a permanent reduction in new venture formation and innovation (Fairlie 2020). For instance, the 2009-2010 global financial crisis caused a long-term reduction in USA start-up rates - see the Economic Innovation Group (2019) for an analysis. A number of recent empirical studies have raised the specter of a long-run impact - for instance Sedlacek and Sterk (2020) estimated that job losses in the USA could be permanent and cumulative and could, over the ten year period 2020 to 2030, exceed 10 million. And the OECD 2020, p.3) issued a concern that the reduction in start-ups will have effects lasting for more than a decade, finding from empirical estimates that “a 20 percent decline in the number of new firms - a drop similar to the one experienced during the global financial crisis - leads to an employment loss of 0,7 percent of aggregate employment 3 years after the shock, and still of 0,5 percent 14 years after.” And if the Great Depression of the 1930s is a benchmark, then technological innovation may be negatively affected for a long time. Babina et al. (2020) found for instance that after the Great Depression patenting declined significantly in counties in the USA that experienced bank distress and moreover that the effects were persistent, concluding that “even though the crisis itself was relatively short-lived, the effects on technological entrepreneurship appear to be permanent—lasting for every decade for the next 70 years” (Babina et al. 2020, p.3).

It is not only that the reduction in start-up rates, firm exists, and innovation will likely have persistent effects, but that the adverse distributional consequences of the pandemic, both for disparity between individuals and countries, and in terms of worsening market concentration, will depress new firm entry and innovation over the longer run - even in spite of short-term increases in start-up rates that may be possible. These distribution consequences were discussed in detail in the previous section. Here we can add that the responses of government to the COVID-19 pandemic can actually worsen these long-run outcomes. As Naudé (2020a) pointed out, the crisis could cause government spending and the share of the government in the economy to crowd out the private sector, including private sector innovation. He refers to the *The Economist* (26th March 2020)^9^ which warned that over “the long term, a vast and lasting expansion of the state together with dramatically higher public debt is likely to lead to a lumbering, less dynamic kind of capitalism.”

*The Economist* (26th September 2020) further argued^10^ in this regard that the wide but selective support by governments to prevent firms, especially in advanced economies, from closing, will “make killing zombie firms off harder,” thus contributing to slower productivity growth and more difficult entry for new firms - see also McGowan et al. (2017). This will compound the problem of already weak innovation in the West. For example, Van Reenen (2019, p.126) pointed out that “the United States spends roughly 240 billion less per year on R&D than it did at its peak.” Naudé and Nagler (2021) documented the decline in innovation in Germany, where most innovation today is done by a few large firms. More generally, Gordon (2012), Cowen (2016) and Erixon and Weigl (2016) documents the pervasive decline in innovation in West since around the 1970s, which Ridley (2020) describes as an “innovation famine.”

## Theoretical Conjectures

In the previous section we argued that the economic shock induced by the non-pharmaceutical interventions (NPIs) aimed at containing the virus that causes COVID-19, is predominantly a demand-shock (also termed a Keynesian supply shock). In a number of recent papers we introduced and elaborated on the idea of demand constrained growth, see e.g. Gries (2018), Gries and Naudé (2018), and Gries and Naudé (2020). Herein we modelled the mechanisms through which a lack of aggregate demand could lead to a reduction in entrepreneurship, as reflected in new venture creation, the implementation of new innovations, and investment.

These are relevant for tracking the consequences of the COVID-19 demand shock. For instance, Gries and Naudé (2020) show that if entrepreneurs cannot sell all their potential production in the market, due to aggregate market conditions and idiosyncratic firm characteristics, they will channel some resources away from production into reducing the mismatch between what they can produce and what is taken up by the market. This can lead to a permanently lower equilibrium growth rate, if the nature of growth is such that the benefits of growth accrues disproportionately to agents with a lower propensity to consume. This could for instance be the case if entrepreneurs bring new technological innovations that can substitute for labor, to market. A demand-shock such as COVID-19, which is characterised by a drop in aggregate demand as well as a redistribution of income towards households with a lower propensity to consume (Palomino et al. 2020), perfectly fits the criteria in the Gries and Naudé (2020) model for leading to permanent reduction in entrepreneurship, innovation and economic growth.

In these endogenous growth models of Gries and Naudé (2018) and Gries and Naudé (2020) where these permanently reduced equilibrium growth paths are derived, the focus is on the general equilibrium macro-mechanisms between demand and growth so that hence, the micro behaviour of agents, such as entrepreneurs, are treated in a somewhat simplified manner. In this section, given that our interest in this paper is on providing a theoretical model to understand the relationship between extreme events such as COVID-19 and entrepreneurship, we provide a more detailed elaboration of the entrepreneur, in particular their role in bringing new innovation to market. This emphasis is, in light of the literature review, due to our concerns that lasting impacts of the COVID-19 pandemic may be due to its impact on innovative entrepreneurship. Departing from our earlier model that aimed to understand entrepreneurship during the global financial crisis of 2009-2010 (Gries and Naudé 2011) we illustrate that entrepreneurial innovation depends not only on new ideas and technology, but also on the market environment. In particular, market demand plays a crucial role. Because of this emphasis, we provide a partial equilibrium model for tractability and clarity and leave its extension to a general equilibrium context for a future exercise.

### The entrepreneurial opportunity

We start the presentation of our model with the entrepreneurial entry decision. We define entrepreneurs as individuals who recognize and exploit opportunities for profitable business, as described by Kirzner (1973) and Schultz (1975). They may recognize opportunities to produce new variants of services or intermediate inputs for large final-goods producing firms. Each product or service variation has certain properties that make the variation unique compared to that of other already existing products or services. This implies that the utilization of opportunities requires entrepreneurial innovation, and that each entrepreneur is essentially a monopolist^11^.

The demand for intermediate goods from the final-goods producers is $ x=\left (\frac {\alpha }{p}\right )^{\frac {1}{1-\alpha }}$. The entrepreneur recognizes in this an opportunity to provide a unique intermediate good, and will decide whether or not to enter the market by establishing a monopolistic new venture around the possible innovation. This decision depends on the expected profits that may be earned as monopolist. Each future period’s profits will be determined by the price of the product innovation *p*_*x*_ and the operating costs of the new venture *c*, i.e. *π* = (*p* − *c*)*x*. In such a case the expected net present value of the monopolistic new venture will be

$$ EV^{m}\left( \tau \right) =\left( 1-\vartheta \right) {\int}_{\tau }^{\infty }(p-c)xe^{-r^{d}(t,\tau )(t-\tau )}dt $$

where *𝜗*_*x*_ represents the expected rate of business failure after market entry, and $\left (1-\vartheta \right ) $ the expected rate of success. Given the demand function, monopoly profits are maximized by the optimal choice of the intermediate good price *p* as

1 $$ p=\frac{c}{\alpha },  $$

where *α* is the elasticity of production of intermediates in the final goods sector. With the optimal price rule we can also determine total demand for intermediates as $x\left (t\right ) =\left (\frac {\alpha ^{2}}{c} \right )^{\frac {1}{1-\alpha }}L_{Q}$. Also, we can see that each period’s profits will depend on the price of the optimal product innovation (1) and the periodic operating costs *c*. Net periodic profits are given by $\pi =\left (p-c\right ) x$ and hence can be calculated as

2 $$ \pi =\left( \frac{1}{\alpha }-1\right) \alpha^{\frac{2-\alpha }{1-\alpha } }c^{-\frac{\alpha }{1-\alpha }}  $$

This means that following successful market entry, the expected maximum present value of the new venture will be

3 $$ EV^{m}=\frac{\left( 1-\vartheta \right) }{r^{d}}\pi  $$

If this value is positive, the entrepreneur will perceive an opportunity.

### Start-up costs

#### Start-up activities

In order to realize this opportunity, an new venture - a start-up - has to be created. This would require the entrepreneur to incur start-up cost *χ*. Start-up costs consists of three components: start-up labor costs (*χ*_*L*_), start-up purchases of investment costs ($v\dot {N}$), and implementation costs such as legal and administrative costs involved in the start-up ($\bar {\chi }$).

The first component of start-up costs, the labor costs (*χ*_*L*_), are necessary to develop new ideas and blueprints of technologies $\dot {A}$, as well as to place these in the market and match with a buyer, i.e. market development. New ideas and blueprints are developed by research laborers (*L*_*A*_) who aims at incremental innovations by building on existing technologies (*A*). Their new ideas and blueprints are generated according to a very simple R&D production process that can be specified as

4 $$ \dot{A}=aL_{A}.  $$

Often entrepreneurs have engineering skills and can provide this resource themselves. However, entrepreneurs can also hire an engineer to conduct all the R&D needed. In both cases the entrepreneur pays wage *w*_*A*_ for the R&D activity, such that research costs are *w*_*A*_*L*_*A*_.

Additional labor costs during the start-up phase are needed because entrepreneurs cannot be sure what the uptake of their new, innovative products will be beforehand. As such, the number of products that will eventually enter the market $\dot {N}$ will be less than the number of ideas and blueprints generated. We assume that the number of matches *M* of ideas and products that will enter the market is determined by general market conditions, as reflected in the aggregate market demand for good *X*^*D*^ as well as the number of variations $\dot {A}$ of the innovative product that the entrepreneur can generate. Further, *M* is also determined by the efforts of labor employed by the entrepreneur. We incorporate these efforts into our model in the form of labor *L*_*M*_ at wage rate *w*_*M*_. The market *match generating mechanism* can therefore be written as

5 $$ \dot{N}=M=\left( L_{M}X^{D}\right)^{\gamma }\left( \dot{A}\right)^{1-\gamma },  $$

The cost for this matching procedure are *w*_*M*_*L*_*M*_. Hence, total start-up labor costs are *χ*_*L*_ = *w*_*A*_*L*_*A*_ + *w*_*M*_*L*_*M*_.

The second component of start-up costs is the start-up purchases of investment goods *v* which directly relate to each product variation $\dot {N}$ that is established in the market. In total these are $v\dot {N}$.

The third and final component of start-up costs are the diverse costs of establishing the new venture, including costs such as drawing up a business plan, performing and covering the legal and other administrative functions, and others. We denote these cost as $\bar {\chi }.$

Having specified the components of start-up costs, we can now write total start-up costs as

6 $$ \chi =\chi_{L}+v\dot{N}+\bar{\chi}.  $$

Since the prospective entrepreneur is assumed to have no present income or accumulated savings, the start-up costs *χ* must be externally financed. The loan rate is denoted by *r*^*l*^. To simplify, we assume a firm that revolves loans infinitely and services interest only (Ponzi finance is excluded). Denoting the deposit rate *r*^*d*^, the present value of start-up cost, (*V*^*s*^) including finance cost, is

7 $$ V^{s}=\frac{r^{l}}{r^{d}}\left[ w_{A}L_{A}+w_{M}L_{M}+v\dot{N}+\bar{\chi} \right]  $$

#### Generating new technologies

Our model makes a distinction between invention, which results in new ideas and blueprints, and innovation. For an invention to become an innovation, and thus obtain a market value, technology development ($\dot {A}$) and market entry of the newly conceived product variations ($\dot {N}$) needs to be approached as an integrated optimization process. This requires the minimization of all relevant costs, starting from the generation of the first ideas through R&D development, and up to placing the innovative product in the market. Formally, the entrepreneur will choose the optimal resource allocation with respect to *L*_*M*_ and *L*_*A*_ subject to the given number of products that is planned to be placed in the market ($\dot {N}=\dot {N}^{plan}=const.$)

8 $$ \begin{array}{@{}rcl@{}} \underset{L_{M},L_{A}}{\min } &:&V^{s}=\frac{r^{l}}{r^{d}}\left[ w_{A}L_{A}+w_{M}L_{M}+v\dot{N}+\bar{\chi}\right] \\ s.t. &\text{:}&\text{ }\dot{N}=\left( L_{M}X^{D}\right)^{\gamma }\left( \dot{A}\right)^{1-\gamma }\ \ \ \ \text{and \ \ \ }\dot{A}=aL_{A} \end{array} $$

From the solution to this problem the optimal inputs of labor for R&D labor and for the placement of the new product are obtained as

9 $$ \begin{array}{@{}rcl@{}} L_{A}^{\ast } &=&\dot{N}\left( X^{D}\right)^{-\gamma }\left( \frac{w_{M}}{ w_{A}}\frac{\left( 1-\gamma \right) }{\gamma }\right)^{\gamma } \end{array} $$

10 $$ \begin{array}{@{}rcl@{}} L_{M}^{\ast } &=&\dot{N}\left( X^{D}\right)^{-\gamma }\left( \frac{w_{A}}{ w_{M}}\frac{\gamma }{\left( 1-\gamma \right) }\right)^{1-\gamma } \end{array} $$

Using (6) this allows us to write the minimum cost for a given number $\dot {N}$of innovative product varieties planned to be placed in the market as

$$ \chi =w_{A}L_{A}+w_{M}L_{M}+v\dot{N}+\bar{\chi}, $$

as well as to express the total costs of launching the start-up, including the costs of finance, as

11 $$ V^{s\ast }=\dot{N}\left( X^{D}\right)^{-\gamma }\frac{r^{l}}{r^{d}}\chi^{\ast },  $$

with

12 $$ \chi^{\ast }=w_{A}\left( \frac{w_{M}}{w_{A}}\frac{\left( 1-\gamma \right) }{ \gamma }\right)^{\gamma }+w_{M}\left( \frac{w_{A}}{w_{M}}\frac{\gamma }{ \left( 1-\gamma \right) }\right)^{1-\gamma }+v\dot{N}+\bar{\chi}  $$

### Launching the new firm

Once the entrepreneur has spotted a revenue-generating opportunity (“?? ??”) and determined and financed the start-up costs (“Start-up costs”) they will make decision whether or not to launch the new venture, which will only be the case if the firm survives the critical start-up phase. A fundamental feature of entrepreneurship is uncertainty. In our literature review, we stressed that extreme events such as the COVID-19 pandemic results in much higher uncertainty, which could amongst others cause households to reduce their mobility and consumption. We denote probability of survival during the start-up phase*p*_*M*_and assume that this probability is determined by the number of product variations that were able to enter the markets and successfully matched the taste of customers *M*. This is a useful assumption to make in the context of the pandemic, where product-consumer matches will be affected by the imposition of non-pharmaceutical interventions and voluntary restriction of consumers’ mobility. For e.g. following a lockdown, products that may be offered in digital form, may find an easier match with a consumer, whilst non-digital products may not.

The probability of surviving the start-up phase therefore is

13 $$ p_{M}=1-e^{-M}  $$

Given *p*_*M*_ and the present value of the expected profit as calculated in Eq. 3, we can now determine the expected present value of an entrepreneurial new venture that includes the start-up phase. We refer to this as the expected gross value of entrepreneurial market entry. It can be written as

14 $$ EV^{g}=p_{M}EV^{m}=p_{M}\left( 1-\vartheta \right) \frac{\pi }{r^{d}}.  $$

Ultimately, the entrepreneur is interested not so much in the gross, but in the net value of market entry. The net value of market entry *EV*^*n*^ is the expected gross value of the project *EV*^*g*^ minus the costs of the entry phase *V*^*s*^, including the development of the technology^12^ (for instance in moving a product online or digitizing a service), planning, and preparing market entry

15 $$ EV^{n}=EV^{g}-V^{s}  $$

As a result, the entrepreneur maximizes the *net present value of the business ventureEV*^*n*^ by choosing the optimal number of innovative goods to bring to the market

$$ \underset{M}{\max }:EV^{n}=\left( 1-e^{-M}\right) \left( 1-\vartheta \right) \frac{\pi }{r^{d}}-M\left( X^{D}\right)^{-\gamma }\frac{r^{l}}{r^{d}}\left[ \chi_{L}^{\ast }+vM+\bar{\chi}\right] . $$

From the First-Order Condition (FOC) and by using the Implicit Function Theorem we obtain the optimal number of innovative product varieties the start-up develops and introduces to the market^13^

16 $$ \dot{N}=M^{\ast }=M^{\ast }\left( X^{D},r^{l},\vartheta \right) ,\text{\ \ with }\frac{dF}{dX^{D}}>0,\text{\ \ \ }\frac{dF}{dr^{l}}<0,\text{\ \ \ } \frac{dF}{d\vartheta }<0  $$

With the optimal number of innovative products the probability of surviving the start-up phase is

17 $$ p_{M}^{\ast }=1-e^{-M^{\ast }},  $$

and the maximum net present value of the business venture is

18 $$ EV^{n\ast }=\frac{1}{r^{d}}\left[ p_{M}^{\ast }\left( 1-\vartheta \right) \pi -M^{\ast }\left( X^{D}\right)^{-\gamma }r^{l}\chi^{\ast }\right] .  $$

As long as there is no stationary state, the new market entrants realize a net rent. Ultimately however, in a competitive steady state equilibrium, the net present value of the business venture is *EV*^*n*∗^ = 0. Thus, the periodic monopoly rents will eventually fully accrue to the entrepreneur as income, and under competitive conditions will be used to finance the start-up costs. From this we can determine the loan rate that such projects are able to pay as

19 $$ r^{l}=\frac{p_{M}^{\ast }\left( 1-\vartheta \right) (1-\alpha )}{\chi^{\ast }}\alpha \left( \frac{\alpha }{c}\right)^{\frac{\alpha }{1-\alpha }}  $$

Having described in the sub-sections above the basic role of entrepreneurship as spotting and using opportunities to create new start-ups and bring new (incrementally) innovative product varieties to the market, in the next section we will use this simple theoretical model to investigate the effects of a COVID-19 like “Keynesian” supply shock.

## Impact of an Extreme Event: Comparative Statics

In “Relevant Literature” we described the essence of the COVID-19 pandemic’s economic consequences as akin to a “Keynesian” supply-shock. The NPIs (lockdowns) which most countries instituted to contain the spread of the virus and prevent the health sector from being overwhelmed, induced at first a supply-shock, which in turn caused a reduction in aggregate demand - which in size and impact exceeded the supply shock. Moreover, the Keynesian supply-shock is termed thus because the nature of the lockdowns in most countries have not affected all sectors, but predominantly those in the services industries where social distancing are difficult to maintain. With one part of the economy more affected than the other, a general increase in demand would be less effective, because part of the economy would not be able respond via increasing supply. Furthermore, the pandemic will in all likelihood raise inequality, which will in term have implications for aggregate demand.

In this section, we use the basic model outlined in “Theoretical Conjectures”, to illustrate the impact of COVID-19’s Keynesian supply-shock on entrepreneurial entry at the individual level (“Impact on individual start-ups”) and on the aggregate innovation rate (“Aggregate impact”), and to derive possible policy recommendations for stimulating recovery (“Supportive policies”). Our model’s comparative statics shows in this section that entrepreneurship recovery after COVID-19 will benefit substantially from aggregate demand-side and complimentary support measures, and moreover that over the short-term an increase in innovation-driven start-up rates is possible.

### Impact on individual start-ups

When confronted with a negative shock to aggregate demand for intermediate goods (i.e. *dX*^*D*^ < 0), as in the case of an extreme event such as COVID-19, the individual start-up entrepreneur will need to spend more on innovation to place the same amount of new product varieties as before. This is because the shortage in aggregate demand will require more labor inputs in order to match the unique new product varieties to reduced demand, as well as more labor inputs for R&D to result in new ideas that may find a buyer (as long as relative wages do not change). This can be seen from that fact that

20 $$ \frac{dL_{A}^{\ast }}{dX^{D}}=-\gamma \left( X^{D}\right)^{-\gamma -1}\dot{N }\left( \frac{w_{A}}{w_{M}}\frac{\gamma }{\left( 1-\gamma \right) }\right)^{1-\gamma }<0  $$

21 $$ \frac{dL_{M}^{\ast }}{dX^{D}}=-\gamma \left( X^{D}\right)^{-\gamma -1}\dot{N }\left( \frac{w_{A}}{w_{M}}\frac{\gamma }{\left( 1-\gamma \right) }\right)^{1-\gamma }<0  $$

Total innovation costs per new product variety good that enters the market will increase since

$$ d\frac{V^{s\ast }}{M}=-\gamma \left( X^{D}\right)^{-\gamma -1}\frac{r^{l}}{ r^{d}}\chi^{\ast }dX^{D}>0 $$

when *dX*^*D*^ < 0.

This result is interesting, because it differs from the standard result of perfect market models, where the cost of innovation is determined only by the cost of the inputs used. In contrast, in our model where the there is no perfect market match for new product varieties (e.g. digital or online based products), the demand side interacts with the costs of innovation, so that a reduction in demand will require a rise in costs.

As a result of this rise in the cost of innovation, a negative demand shock (*dX*^*D*^ < 0) will result in a reduction in the entrepreneur’s probability of surviving the start-up phase, since

22 $$ dp_{M}^{\ast }=e^{-M}\frac{\gamma }{X^{D}}dX^{D}<0  $$

It will also reduce the expected net present value of the new start-up^14^

23 $$ dEV_{x}^{n\ast }=\gamma \left( X^{D}\right)^{-\gamma -1}M^{\ast }\frac{r^{l} }{r^{d}}\chi^{\ast }dX^{D}<0,  $$

The implication is that the likelihood that individual entrepreneurs will proceed with a new start-up will decline following a negative shock to aggregate demand. This is indeed what we see following the COVID-19 shock. For example, early empirical evidence from New York State indicates that high-propensity business applications^15^ declined by 50 percent in March-April 2020 over the same period in 2019 (Sedlacek and Sterk 2020). Fairlie (2020) records a decline of 22% in the number of active business owners in USA between February and March 2020. Other authors have also noted a sharp increase in the expected number of firm failures and delayed or cancelled start-ups, see e.g. Bartik et al. (2020) and Bosio et al. (2020).

There is however one automatic “stabilizer” at work here, in that after a demand shock of *dX* < 0, the interest rate at which the entrepreneur can obtain a loan to cover start-up costs, will be lower. This could be, amongst others, due to a “flight to safety” and an increase in precautionary savings (Caballero and Farhi 2018). This partial effect (our model is not a general equilibrium model) can be seen from the following.

24 $$ dr^{l}=\left[ \frac{dp_{M}^{\ast }}{dX^{D}}-\frac{1}{\chi^{\ast }}\frac{ d\chi^{\ast }}{dX^{D}}\right] \frac{\left( 1-\vartheta \right) }{\chi^{\ast }}(1-\alpha )\alpha \left( \frac{\alpha }{c}\right)^{\frac{\alpha }{ 1-\alpha }}dX^{D}<0.  $$

The reduction in the interest rate that accompanies a negative demand shock will be beneficial for entrepreneurs in light of the fact that their start-up costs will decrease- it may limit the extent to which the rate of start-ups will decline in the wake of the shock. The net impact on the likelihood that the individual will start-up a new venture is theoretically ambiguous, and ultimately an empirical matter. As we will show below (see also Fig. 1) it has indeed been the case that start-up rates increased in a number of countries.

### Aggregate impact

In this sub-section we move from the level of the individual entrepreneur to determine the consequences for the aggregate economy. We start by denoting the total number of innovative products (they are identical to the number of new start-ups) by $\dot {N}$. Because of our assumption that all new innovative products are variations and only incrementally differentiated (i.e. none are radical innovations) we implicitly assume that they have identical economic properties. Hence, they will all have the same expected net value $\left (EV^{n}\right )^{\ast }$.

However, unlike their incremental innovations, entrepreneurs are not similar with respect to risk-taking behavior, in fact they are heterogeneous in this respect. Thus different entrepreneurs will have different risk-return trade-offs, i.e. demand different expected net values from market entry to compensate for risk. So for instance in the case of entrepreneur *i*, it will only be if the expected net value of market entry $\left (EV^{n}\right )^{\ast }$ exceeds their particular threshold $\left (EV^{n}\right )^{i}$ that they would start-up the new venture and launch their innovation activities. In other words, only if

25 $$ EV^{n\ast }>EV^{ni}  $$

will they initiate entrepreneurial venture, otherwise there will be no market entry, and no innovation. We assume that required threshold for market entry indicated by $\left (EV^{n}\right )^{i}$ is normally distributed with parameters *μ* and *σ*^2^, in other words:

$$ \left( EV^{n}\right)^{i}\sim \mathcal{N}(\mu ,\sigma^{2}), $$

With this distribution of ${p_{M}^{i}}$ we can determine total market entry from the probability of ${p_{M}^{i}}<p_{M}^{\ast }$. This probability is described by the following distribution function:

$$ P(EV^{ni}<EV^{n\ast })=F(EV^{n\ast })=\frac{1}{2}\left( 1+erf\left( \frac{ EV^{n\ast }-\mu }{\sqrt{2\sigma^{2}}}\right) \right) $$

Where *erf*(*x*) is the Gauss error function with $erf(x)=\frac {1}{\sqrt {\pi }} {{\int \limits }_{0}^{x}}e^{-t^{2}}dt$. As each probability *EV*^*ni*^ relates to one innovation and one entrepreneur, *F*(*EV*^*n*∗^) is the share of all entrepreneurs planning market entry that indeed initiate their business venture.

If a demand shock leads to a change of *EV*^*n*∗^ such that $\frac { dEV^{n\ast }}{dX^{D}}>0$ (see Eq. 23) we can determine the share of start-up ventures by using the density function

26 $$ F^{\prime }(EV^{n\ast })=f(EV^{n\ast })=\frac{1}{\sqrt{2\pi \sigma^{2}}} \exp \left( \frac{(EV^{n\ast }-\mu )^{2}}{2\sigma^{2}}\right) .  $$

That is, using (see Eqs. 23 and 26 we can see that with *dX*^*D*^ < 0 in aggregate start-up ventures decline by

27 $$ dF=\frac{\partial F}{\partial EV^{n\ast }}\frac{\partial EV^{n\ast }}{ \partial X^{D}}=\frac{1}{\sqrt{2\pi \sigma^{2}}}\exp \left( \frac{ (EV^{n\ast }-\mu )^{2}}{2\sigma^{2}}\right) \frac{\partial EV^{n\ast }}{ \partial X^{D}}dX^{D}>0  $$

Finally, the number of overall innovative products also crucial depends on demand (*X*^*D*^). According to Eq. 16 innovative products launched by each start-up firm is $M^{\ast }=M^{\ast }\left (X^{D},r^{l},\vartheta \right ) ,$ such that a negative demand shock *dX*^*D*^ < 0 reduces the number of new innovative products of each start-up by

28 $$ d\dot{N}=dM^{\ast }=\frac{\partial M^{\ast }}{\partial X^{D}}dX^{D}<0.  $$

In addition the expected number of firms that indeed enter the market with their new innovation is also affected as we derived in Eq. 27. So both components add to the effect on aggregate innovation. The expected total number of innovative products $E\left [ \dot {N}\right ] $will be $E\left [ \dot { N}\right ] =\dot {N}F(EV^{n\ast })$ and the effect of the demand in the moment *dX*^*D*^ < 0 on expected innovation is

$$ dE\left[ \dot{N}\right] =\left[ \frac{\partial M^{\ast }}{\partial X^{D}}+ \dot{N}\frac{\partial F}{\partial EV^{n\ast }}\frac{\partial EV^{n\ast }}{ \partial X^{D}}\right] dX^{D}<0 $$

However, if demand is buoyed by government financial support and related measures to reduce uncertainty (*dX*^*D*^ > 0) then clearly, the rate of innovation (and hence of innovative start-ups) ***will increase not only on the individual level, but in aggregate***. This is consistent with available empirical evidence on innovation rates following major crises, see for instance Babina et al. (2020) and Ionescu-Somers (2020).

It is moreover also consistent with evidence from COVID-19 pandemic which suggests that in some countries, notably the USA and France, start-up rates of innovative firms have after taking an initial dip in 2020, actually began rising towards the middle of the year (Romei 2020). For instance, as the USA Census Bureau documents (see https://www.census.gov) in the USA business formation applications for high-propensity start-ups have increased substantially since around July 2020.

High-propensity start-ups are defined as “Business Applications (BA) that have a high-propensity of turning into businesses with payroll”, and is determined by the USA Census Bureau based on “the characteristics of applications revealed on the IRS Form SS-4 that are associated with a high rate of business formation.” These are therefore start-ups that are most likely to create jobs for others and to be undertaken in view of an opportunity, and not out of necessity. As such, high-propensity start-ups may be a measure of start-ups undertaken to bring an innovation to market. Romei (2020) discuss some of the drivers of such innovation during the COVID-19 pandemic, such as new opportunities in online retail, as well as provision of “logistics, home delivery of goods and services, technology, digital wellness and fitness”.

Figure 1 depicts high-propensity start-up rates in the USA between January 2019 and December 2020. It shows that between December 2019 and April 2020, start-up rates as measured by high-propensity business applications declined by 30% - but then recovered as government financial support as well as the opportunities implied by the Keynesian supply-shock took effect, with start-up rates rising between April 2020 and July 2020 by 134%. By the end of 2020, high-propensity start-ups were back at their December 2019 level.

Further, there may be an acceleration of certain types of innovations in response to an extreme event or disaster. The model set out here implies that an extreme event can create demand pull effects towards these innovations. Take as an example the disaster at Fukushima in 2011. This resulted in a signal that accelerated the global shift of demand towards renewable energy - Germany for instance decided to completely withdraw from nuclear energy and further promoted subsidized renewable energy. This led to an expansion of the demand for renewable energy and promote further innovations in this sector. In this light, our model reflects the idea that the level and direction of innovations is not the result of a random engineering process, but that it is the result of engineering efforts that are directed by a particular level and structure of the demand for these innovative goods.

### Supportive policies

In the previous sections we identified the mechanisms which affect new entrepreneurial venture start-ups and innovation. Our model’s comparative static results indicated that extreme events will have, in the absence of government interventions, and on balance, negative impacts on entrepreneurship and innovation - although over the short-term due to the heterogeneous impacts of such events may also have positive impacts on innovation. This is a conclusion that is supported by a reading of the relevant literature (see “?? ??”).

Negative effects on entrepreneurship and innovation, as reflected in our model, can be countered by policies. In the case of COVID-19 financial support from governments, where it has been forthcoming, have been found to make a difference to a whole range of outcomes. For example, in the case of poverty in the USA, Martin et al. (2020, p.471) found that “government benefits [...] decrease the amplitude and duration of the crisis. In likely scenario of a 3-month crisis period, the increase in poverty can be limited to 19% (from 17.1% at pre-crisis), and the average time of recovery almost halved to 6.7 months.” Similarly to decreasing the amplitude and duration of poverty from the pandemic, government financial support to households and firms can decrease the amplitude of business failures and the duration of the contraction in start-ups. Given the asymmetric and heterogeneous impacts of the Keynesian supply-shock and the distributive impacts of the pandemic, the joint effect can even be that after an initial contraction in start-up activity, that such activity can quickly rebound to pre-pandemic rates - as we already illustrated in the case of innovation-driven start-ups in the USA during the pandemic (see Fig. 1). This will be as government benefits together with distributive impacts will improve the expected net present value of many start-up ventures following the initial pandemic shock.

Given this possibility, in this section we consider the maximum net present value of the project and describe what kind of financial support *𝜃* a government can offer to compensate existing and potential future entrepreneurs from the demand shock, as well as the diminished probability of successful market entry. Moreover, in the presence of a Keynesian supply-shock, entrepreneurs need to be compensated from the additional effects of the lockdown measures on survival and new market entry, due to the fact that some sectors (for example hospitality or events) will not be able to benefit from a general expansionary demand stimulus, including fiscal and/or interest rate policies - because their ability to increase supply and get back to normal is administratively limited - as we observe in various parts of the service sector.

The simple model outlined in “Theoretical Conjectures” easily lends itself to illustrate that three direct measures can be undertaken to compensate entrepreneurship during a Keynesian supply shock. One is a direct subsidy for losses in own revenues (*𝜃*_*π*_ - which can also be seen as government acting as a consumer of last resort (Saez and Zucman 2020). Such a consumer-of-last resort function is akin to insurance and will have the effect to compensate for the higher expected failure rate under the lockdown. A second supportive policy *𝜃*_*χ*_ is a direct subsidy to start-ups, especially given as it has been noted that fiscal support during COVID-19 by most countries tend to neglect start-ups (Brown and Rocha 2020; Kuckertz et al. 2020). A third supportive policy, *𝜃*_*r*_ is a subsidy on loan liabilities, which is important given the demand spillover effects that characterise a Keynesian supply-shock - the subsidy on loan liabilities essentially counteracts any unanticipated loss in revenues.

$$ \begin{array}{@{}rcl@{}} EV^{n} &=&\frac{1}{r^{d}}\left[ p_{M}^{\ast }\left( 1-\vartheta \right) (p-c+\theta_{\pi })x-\left( r^{l}-\theta_{r}\right) \chi^{\ast }+\theta_{\chi }\right] , \\ \frac{dEV^{n}}{d\theta_{\pi }} &=&\frac{p_{M}^{\ast }\left( 1-\vartheta \right) }{r^{d}}>0,\quad \frac{dEV^{n}}{d\theta_{\chi }}=1,\quad \frac{ dEV^{n}}{d\theta_{r}}=\chi_{x}^{\ast } \end{array} $$

All these subsidies are suitable to improve the expected net present value of the business venture and compensate for the overall negative impact which is described in Eq. 27. Note that while direct subsidies to start-ups will improve the rate of start-ups unambiguously in our model, this does not suggest that government should always provide this - apart from the political economy arguments against such possibly distorting subsidies, government resources may be limited and prioritised on health spending and support for households and existing firms. These latter expenditures are of course, as our model showed, consistent with increasing aggregate consumption and reducing the uncertainties surrounding the pandemic, and as such would have a positive impact on individual and aggregate start-up rates. Due to space limitations we leave the derivation of the comparative statics of these supportive policies as a exercise for the eager reader.

## Concluding Remarks

In 2011, in the wake of the global financial crisis, we bemoaned the lack of research on the links between extreme events and entrepreneurship - see Gries and Naudé (2011). Since then, there has been a significant increase in scholarly work on the topic. The bulk of this work however, useful as it is, is empirical and descriptive. In this paper we contributed to rectify this gap in the literature by providing some simple theoretical conjectures so as to enrich the set theoretical models available with which to explain the impacts of extreme events, and specifically the impact of a pandemic such as COVID-19, on entrepreneurship.

We did so by selectively reviewing the current literature, and departing from our own earlier (2011) work on the impact of the global financial crisis on entrepreneurship (see Gries and Naudé 2011). Moreover, we argued that the particularities of the COVID-19 crisis need to be taken explicitly into account, and that these are different from other extreme events.

A particularity that we considered was the fact that COVID-19 is a “Keynesian” supply-shock, that is a supply-shock which causes a reduction in aggregate demand and where part of the supply response of the economy remains “locked down” (see Guerrieri et al. 2020). In such a context, an aggregate demand expansion, through for instance raising government spending, although useful, will be less effective (the Keynesian multiplier will be smaller) and will need to be complemented by specific measures. Our model showed that these could include direct subsidies for revenue losses, for start-ups and for loan liabilities.

Our model’s comparative statics showed that a negative demand shock, such as being experienced during the COVID-19 pandemic will lead to reduced likelihood that an individual will start-up a new venture, and may reduce the aggregate rate of entrepreneurship and innovation. Over the short-term, in countries where entrepreneurs can respond by developing new digital product and online service varieties, and where fiscal support to households and existing firms are forthcoming, together with adequate and open international transport and logistics services, a Keynesian supply-shock may even stimulate a rise in innovation-driven start-ups. The key therefore to protecting entrepreneurship and innovation during the COVID-19 pandemic is to limit the extent to which aggregate demand declines, to compensate existing entrepreneurs and start-ups from the Keynesian supply-side shock of lockdown measures, to ensure access to ICT services and the effective functioning of transport and logistic systems. There is also, as a role for expansionary demand-side policies such as a fiscal expansion and monetary policy changes, to be complemented by additional measures such as a direct subsidies for losses in own revenues, for start-ups, and for loan liabilities. In our model all three of these specific measures will counter the effects of a Keynesian supply-shock.

Finally, a notable implication from our paper is that recovery of entrepreneurship after COVID-19 will benefit from re-distributive measures - as this will help raise aggregate demand. We have shown, in our survey of the literature, that there are good grounds to be concerned that the pandemic will lead to an increase in income inequality. In a related paper, we specified the links between inequality and aggregate demand in the the context of an endogenous growth model, see Gries and Naudé (2020). Based on this, a re-distributive measure that we can stress here, but however which we have leave to be evaluated through an extension of our modelling framework in future, is to improve diffusion of, and access to digital infrastructures (shown by the COVID-19 pandemic to be inadequate in many countries and regions). This will help reduce the fixed costs to entrepreneurial entry (in effect lowering the productivity barrier to entry), reduce digital gaps (indirectly contributing to reducing inequality and raising aggregate demand), and can moreover contribute towards putting future economic growth on a more environmental sustainable trajectory as well (Piller 2020). An extreme event such as COVID-19 does not need to leave a lasting legacy of destruction: appropriate supportive policies coupled with entrepreneurial ingenuity can facilitate recovery and even to a subsequent growth and innovation boom.

## Appendix:

In “Impact on individual start-ups” we stated that when confronted with a negative shock to aggregate demand for intermediate goods, i.e. *d**X*^*D*^ < 0, the individual start-up entrepreneur will require more labor inputs in order to match their unique products to the reduced demand from the final-goods producer. This can be seen from that fact that

29 $$ \frac{dL_{M}^{\ast }}{dX^{D}}=-\frac{\frac{\partial F}{\partial X^{D}}}{ \frac{\partial F}{\partial R_{M}}}<0  $$

This result can be derived in this appendix by taking the first-order condition (F.O.C.) for maximizing the expected net present value $E{V_{x}^{n}}$ which is

$$ \begin{array}{@{}rcl@{}} \frac{dE{V_{x}^{n}}}{dL_{M}} &=&F=-e^{-L_{M}\left( X\right)^{\gamma }\left( \dot{A}\right)^{1-\gamma }}\left( -\left( X\right)^{\gamma }\left( \dot{A} \right)^{1-\gamma }\right) \frac{\pi_{x}}{r^{d}}-\frac{r^{l}}{r^{d}} c_{M}=0. \\ \frac{d^{2}E{V_{x}^{n}}}{d{L_{M}^{2}}} &=&\frac{dF}{dL_{M}}=-e^{-L_{M}\left( X\right)^{\gamma }\left( \dot{A}\right)^{1-\gamma }}\left( \left( X\right)^{\gamma }\left( \dot{A}\right)^{1-\gamma }\right)^{2}\frac{\pi_{x}}{r^{d} }<0 \end{array} $$

Now, with $\frac {d^{2}E{V_{x}^{n}}}{d{L_{M}^{2}}}<0$ we identify a maximum. Further, we can apply the I.F.Th which requires $\frac {dF}{dL_{M}}\neq 0,$ such that $ 0=F=F\left (L_{M},X\right ) $ implicitly defines a function

$$ L_{M}^{\ast }=L_{M}\left( X,\dot{A}\right) . $$

Further,with

$$ \begin{array}{@{}rcl@{}} \frac{d^{2}E{V_{x}^{n}}}{d{L_{M}^{2}}} &=&\frac{dF}{dL_{M}}=-e^{-L_{M}\left( X\right)^{\gamma }\left( \dot{A}\right)^{1-\gamma }}\left( \left( X\right)^{\gamma }\left( \dot{A}\right)^{1-\gamma }\right)^{2}\frac{\pi_{x}}{r^{d} }<0\qquad and \\ \frac{d^{2}E{V_{x}^{n}}}{dE{V_{x}^{n}}dX} &=&\frac{dF}{dX}=-e^{-R_{M}\left( X\right)^{\gamma }\left( F\left( R_{A}\right) \right)^{1-\gamma }}\left[ -\gamma L_{M}\left( X\right)^{\gamma -1}\left( \dot{A}\right)^{1-\gamma } \right] \left( \left( -\right) \left( X\right)^{\gamma }\left( \dot{A} \right)^{1-\gamma }\right) \\ &&-e^{-L_{M}\left( X\right)^{\gamma }\left( \dot{A}\right)^{1-\gamma }}\gamma L_{M}\left( X\right)^{\gamma -1}\left( \dot{A}\right)^{1-\gamma } \\ &=&-e^{-L_{M}\left( X\right)^{\gamma }\left( \dot{A}\right)^{1-\gamma }} \left[ -\gamma L_{M}\left( X\right)^{\gamma -1}\left( \dot{A}\right)^{1-\gamma }\right] \left[ \left( \left( -\right) \left( X\right)^{\gamma }\left( \dot{A}\right)^{1-\gamma }\right) -1\right] \\ &=&-e^{-L_{M}\left( X\right)^{\gamma }\left( \dot{A}\right)^{1-\gamma }} \left[ \gamma L_{M}\left( X\right)^{\gamma -1}\left( \dot{A}\right)^{1-\gamma }\right] \left[ \left( \left( X\right)^{\gamma }\left( \dot{A} \right)^{1-\gamma }\right) +1\right] <0 \end{array} $$

we obtain:

$$ \begin{array}{@{}rcl@{}} 0 &=&F=F\left( L_{M},X\right) \\ 0 &=&dF=\frac{\partial F}{\partial L_{M}}dL_{M}+\frac{\partial F}{\partial X} dX \\ \frac{dL_{M}}{dX} &=&-\frac{\frac{\partial F}{\partial X}}{\frac{\partial F}{ \partial L_{M}}}<0 \end{array} $$

## Footnotes

1 Note that rising unemployment may push many people into self-employment and hence there may be a rise in the short-term in necessity-motivated start-up rates. Shifts in demand due to lockdowns may also lead to a rise in start-ups in the sectors benefiting. The extent to which start-up rates will decline over the short-term will also depend on government support measures - as we will in fact also illustrate in this paper.

2 By mid-2020 the IMF and OECD predicted that world GDP would contract by respectively 4,9 and 6,0 percent in 2020 (IMF 2020; Boone 2020).

3 We will follow Brück et al. (2011) and use the term “extreme events” in this paper to refer to natural and human-made disasters, such as wars, major political crises, macro-economic shocks and crises, including global financial crises, and of course disasters such as epidemics and pandemics.

4 Aguirre et al. (2020, p.256) suggests that “to prevent future pandemics, the sale of exotic animals be banned at wet markets.”

5 Ionescu-Somers (2020) list a number of examples of innovations that followed on from previous crises and recessions, recalling for example that “wars and crises have stimulated innovation on multiple levels, leapfrogging technological innovation and enabling social empowerment, human rights or environmental movements, and even the creation of multilateral organizations such as the United Nations and the World Health Organization.”

6 As Yoo and Managi (2020) show, the pandemic also imposes a huge economic cost through lives lost, and that lockdown measures, whether voluntary or involuntary, reduces the mortality costs significantly.

7 As Naudé and Cameron (2021) report, the RWI/ISL Container-Throughput Index (which measures goods moving through the world’s ports) indicates that world merchandise trade movements have recovered fully by the end of 2020.

8 Gokmen and Morin (2019) find evidence from studying a number of financial crises between 1973 and 2006 that generally there is overall significant relationship between financial crises and inequality, except in the case of stock market crises in advanced economies, which tend to be followed by periods of declining inequality.

9 See https://www.economist.com/leaders/2020/03/26/the-state-in-the-time-of-covid-19.

10 See https://tinyurl.com/yyc6w8mn

11 Our entrepreneur would thus be the ideal entrepreneur as espoused by Thiel (2015) - avoiding competition by innovating a unique product.

12 We can note here that the cost of digitization, reflected in the cost of computing and the cost of investment in ICT, has declined exponentially, see e.g. Friedman (2016) and Karabarbounis and Neiman (2014). This makes the creation of start-ups based on digital business models much less costly than before, and less costly than bricks-and-mortar based business models.

13 See the A.

14 This result follows from the Envelope Theorem which we apply to Eq. 18.

15 High-propensity business applications are defined by the US Census as new business applications that “have a high propensity of turning into businesses with payroll. Thus, these type of start-ups are a close empirical counterpart to our notion of entrepreneurship.
